# Congenital Titinopathies Linked to Mutations in *TTN* Metatranscript-Only Exons

**DOI:** 10.3390/ijms252312994

**Published:** 2024-12-03

**Authors:** Aurélien Perrin, Rocio Garcia-Uzquiano, Tanya Stojkovic, Céline Tard, Corinne Metay, Anne Bergougnoux, Charles Van Goethem, Corinne Thèze, Marion Larrieux, Héloise Faure-Gautron, Jocelyn Laporte, Guillaume Lefebvre, Martin Krahn, Raul Juntas-Morales, Titin’s Network Collaborators, Michel Koenig, Susana Quijano-Roy, Robert-Yves Carlier, Mireille Cossée

**Affiliations:** 1 Laboratoire de Génétique Moléculaire, Centre Hospitalier Universitaire de Montpellier, 34093 Montpellier, France; anne.bergougnoux@inserm.fr (A.B.); c-vangoethem@chu-montpellier.fr (C.V.G.); corinne.theze@inserm.fr (C.T.); marion.larrieux@ext.inserm.fr (M.L.); heloise.faure-gautron@inserm.fr (H.F.-G.); michel.koenig@inserm.fr (M.K.); 2 PhyMedExp, INSERM, CNRS, Université de Montpellier, 34093 Montpellier, France; 3 AP-HP, GHU Université Paris-Saclay, Neuromuscular Center, Child Neurology and ICU Department, Raymond Poincare Hospital, 92380 Garches, France; rocio.garciauzquiano@aphp.fr (R.G.-U.); susana.quijano-roy@aphp.fr (S.Q.-R.); 4 AP-HP, Centre de Référence des Maladies Neuromusculaires Nord/Est/Île-de-France, Sorbonne Université, Hôpital Pitié-Salpêtrière, 75013 Paris, France; stojkovic.tanya@aphp.fr; 5 Département de Neurologie et des Troubles du Mouvement, U1172, Centre Hospitalo Universitaire (CHU) de Lille, CT, Centre de Référence des Maladies Neuromusculaires Nord/Est/Île-de-France, 59000 Lille, France; celine.tard@chu-lille.fr; 6 AP-HP, UF Molecular Cardiogenetics and Myogenetics, Sorbonne Université and Sorbonne Université UPMC Paris 06, Inserm UMRS974, Research Center in Myology, Pitié-Salpêtrière Hospital, 75013 Paris, France; corinne.metay@aphp.fr; 7 Institut de Génétique et de Biologie Moléculaire et Cellulaire (IGBMC), Inserm U1258, CNRS UMR 7104, Université de Strasbourg, 67400 Illkirch, France; jocelyn@igbmc.fr; 8 Service d’Imagerie Musculo-Squelettique, CCIAL, CHU de Lille, Rue Emile Laine, 59037 Lille, France; guillaume.lefebvre@chu-lille.fr; 9 INSERM, Marseille Medical Genetics, U1251, Aix-Marseille Université, 13385 Marseille, France; martin.krahn@univ-amu.fr; 10 Département de Génétique Médicale, Hôpital Timone Enfants, APHM, 13385 Marseille, France; 11 Neurology Department, Vall d’Hebron University Hospital, 08035 Barcelona, Spain; rjuntas@vhebron.net; 12 U1179 INSERM-UVSQ, Université de Versailles, 78180 Montigny, France; robert.carlier@aphp.fr; 13 AP-HP, GHU Université Paris-Saclay, DMU Smart Imaging, Radiology Department, Raymond Poincaré Teaching Hospital, 92380 Garches, France

**Keywords:** congenital titinopathies, myopathy, genetics, neurology, arthrogryposis

## Abstract

Congenital titinopathies reported to date show autosomal recessive inheritance and are caused by a variety of genomic variants, most of them located in metatranscript (MTT)-only exons. The aim of this study was to describe additional patients and establish robust genotype–phenotype associations in titinopathies. This study involved analyzing molecular, clinical, pathological, and muscle imaging features in 20 patients who had at least one pathogenic or likely pathogenic *TTN* variant in MTT-only exons, with onset occurring antenatally or in the early postnatal stages. The 20 patients with recessive inheritance exhibited a heterogeneous range of phenotypes. These included fetal lethality, progressive weakness, cardiac or respiratory complications, hyper-CKemia, or dystrophic muscle biopsies. MRI revealed variable abnormalities in different muscles. All patients presented severe congenital myopathy at birth, characterized by arthrogryposis (either multiplex or axial–distal) or neonatal hypotonia in most cases. This study provides detailed genotype–phenotype correlations in congenital titinopathies caused by mutations in MTT-only exons. The findings highlight the variability in clinical presentation and the severity of phenotypes associated with these specific genetic alterations. RNA-seq analyses provided valuable insights into the molecular consequences of *TTN* variants, particularly in relation to splicing defects and nonsense-mediated RNA decay. In conclusion, this study reinforces the genotype–phenotype correlations between congenital myopathies and variants in *TTN* MTT-only exons, improves their molecular diagnosis, and provides a better understanding of their pathophysiology.

## 1. Introduction

Titinopathies are a group of myopathies caused by defects in the titin gene (*TTN*—*OMIM*-*188840*) localized on chromosome 2q31.2. The condition’s phenotypes range from fetal lethality to mild weakness of onset at the adult age. They may show arthrogrypotic signs, motor delay, or late limb-girdle weakness, require respiratory management, and/or present with life-threatening cardiac disease [1]. In the case of congenital titinopathies, the first signs are present antenatally, at birth, or in the first months of life. In mild forms, patients are often diagnosed at a later stage and early symptoms are discreet. At the molecular level, mutations can have recessive or dominant effects. Titin is encoded by the *TTN* gene of 364 exons and codes for the giant muscle protein titin [2]. It spans half of a sarcomere, the functional contractile unit of muscle cells, from the Z line to the M-band. Titin plays a structural role and is responsible for the elasticity of sarcomeres in skeletal and cardiac muscle. This elasticity is due to the PEVK domain, a region of high sequence homology between exons, and named for its high proportion of Pro-Glu-Val-Lys amino acids [3,4]. The PEVK domain binds many different proteins resulting in its involvement as a component in signal transduction [5].

The *TTN* gene is subject to extensive alternative splicing. As a result, it produces tissue-specific isoforms, most notably in skeletal and cardiac muscle. The splicing pattern can also vary during development. The theoretical isoform that includes all 363 coding exons is called the *TTN* metatranscript. Some exons of the metatranscript are systematically spliced out in skeletal muscles after birth (N2A isoform), while others are spliced out to various degrees [6]. Exons not or partially included in any of the cardiac and skeletal muscle main isoforms are defined as “metatranscript-only exons” (MTT-only exons). The effect of a potentially pathogenic variant could have different consequences, whether it is in an MTT-only exon or in a constitutively expressed exon. Among these MTT-only exons, there is a particular domain consisting of three blocks of repeated exons. The sequence homology is greater than 99%, located between exons 173 and 199 in the PEVK domain.

To date, all pathogenic *TTN* variants that have been described as associated with early-onset or congenital myopathies show recessive inheritance. The first article to describe this genotype–phenotype association, Fernandez-Marmiesse et al. [7], reported the case of a newborn with a multiple arthrogryposis pattern. This patient presented with a phenotype of severe hypotonia associated with a homozygous frameshift variant in the MTT-only exon 197 (p.(Lys12887Asnfs*6)). The authors propose that protein defects in the fetal period were responsible for the severe clinical picture at birth, whereas the stability of the disease after birth is thought to be due to the presence of a normal protein expressed by the N2A isoform [7]. Similarly, Oates et al., described in their study that, among 30 patients with recessive congenital titinopathy, 10 had one or both pathogenic variants localized in an MTT-only exon [8]. This study suggested that these mutations result in a muscle developmental defect and that the severity of muscle damage subsequently decreases due to the non-expression of the mutated exon after birth [8]. In total, 55 patients were described in the literature as having arthrogryposis associated with at least one variant in MTT-only exons [7,8,9,10,11,12,13,14,15,16,17,18]. Thus, mutations in MTT-only exons could disrupt titin’s elasticity, resulting in skeletal muscle defects in muscle development that manifest as congenital myopathies such as arthrogryposis.

Although the role of titin in muscle elasticity is well established, the specific impact of mutations in MTT-only exons on early-onset myopathies remains underexplored. This study aims to analyze the phenotypic and genotypic spectra of additional pediatric patients and to report adult patients, who are rarely described in the literature. In this study, we analyzed the molecular, clinical, pathological, and muscle imaging features of 20 patients with pathogenic or likely pathogenic *TTN* variants. In all, at least one of the variants was in an MTT-only exon, which was an inclusion criterion.

## 2. Results

### 2.1. Global Cohort

**Figure 1:**
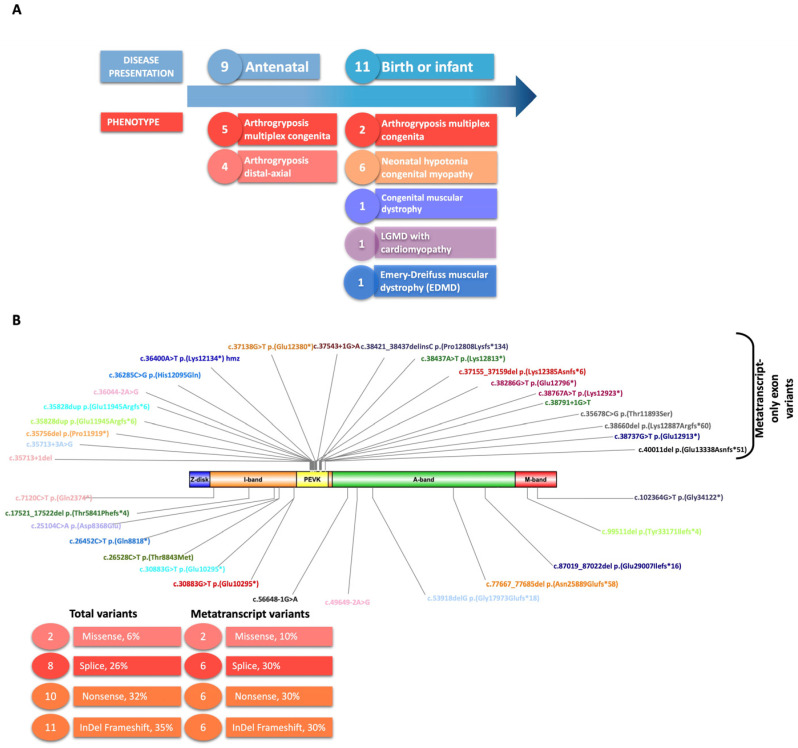
Cohort description and location of variants on the *TTN* gene. (**A**) Clinical presentation. (**B**) Genomic variants mapped on the *TTN* gene; colors are attributed to each patient. Variants described in the upper panel are metatranscript-only exon variants and, in the lower panel, constitutively expressed exons.

The cohort consisted of 20 patients from 17 unrelated families. There were 11 males, 6 females, and 3 fetuses. Of the 17 living patients analyzed, 11 were adults and 6 were between 1 and 17 years old at the time of the study. Presentation of the disease was antenatal (n = 7), at birth (n = 12) or during a very early childhood period (n = 1) (Figure 1A). 15 patients out of 20 presented signs of immobility at early stages of the fetal life, mainly arthrogryposis multiplex congenita (n = 7) and distal–axial arthrogryposis (n = 4) (Figure 1A, Table 1 and Table S1).

We identified 31 *TTN* variants, 30 of which were pathogenic and 1 of uncertain significance (Table S2). All patients had at least one variant in an MTT-only exon (inclusion criterion). Among the 31 variants, most of them were truncating (11 frameshift and 10 nonsense variants), 8 were in consensus splice sites and 2 were missense variants. All the 20 variants in an MTT-only exon were in the PEVK domain at the I-band (Table 1 and Table S2, Figure 1B). The location of the variants in the constitutively expressed exons was not specific to a particular domain (Figure 1B).

### 2.2. Clinical Data

Among the twenty patients (Table 1 and Table S1), the following phenotypes were identified: arthrogryposis, congenital myopathy, and muscular dystrophy.

#### 2.2.1. Arthrogryposis (11 Patients)

Seven patients presented with arthrogryposis multiplex congenita (AMC). In three of the cases, a prenatal diagnosis led to medical pregnancy termination (P2, P6, P8). The remaining four patients (P3, P4, P5, P7) were born and showed a stable–improving clinical course without respiratory failure and achieved motor milestones. Four patients had axial–distal arthrogryposis (P9, P10, P11, P12). Most of them (P10 and P12) showed a stable course or slowly improving motor milestones, and one (P11) had an evolutive phenotype with loss of ambulation and scoliosis. P10 was thoroughly described in a previous case report [18].

One of these 11 patients (P9) was the most severe case, with severe congenital hypotonia, distal malformations (club feet, arachnodactyly), dysmorphic features (microretrognathia, ogival palate), facial weakness, and suction and swallowing difficulties requiring a gastrostomy. The patient developed spinal stiffness, periscapular amyotrophy, and pes cavus. The patient was weaker in the upper limbs, was able to bend arms against gravity, but had forearm extensor weakness with drooping hands. The evolution of the pathology was favorable: gastrostomy was closed before 10 years and nocturnal ventilation was stopped in early childhood.

#### 2.2.2. Congenital Myopathy (Four Patients)

All four patients presented with hypotonia in the neonatal period and showed motor weakness, but did not show joint contractures or scoliosis. P15 and P16 showed a favorable course. P15 was able to walk without too much delay, climb stairs soon afterwards during infancy, and run during childhood, with a slight weakness of the waist and proximal part. P16’s condition was more severe, and she was wheelchair-bound during childhood, started to walk with assistance during adolescence, and walked without assistance indoors at adulthood. Muscle atrophy was predominant on the posterior thigh and the anterior leg during her last consultation.

P17 and P18 were sisters (Family 16), and both acquired walking in the first two years of life. They showed a less favorable course, with loss of motor function and respiratory insufficiency, which did not require ventilatory support.

#### 2.2.3. Muscular Dystrophy Phenotype (Five Patients)

All acquired walking and had high creatine kinase (CK) levels or dystrophic biopsy, and their course was progressive, with variable contractures and cardiac involvement. P1 had a limb-girdle phenotype (LGMD). He developed progressive weakness—predominantly in the proximal and girdle muscles—as well as high CK and myocardiopathy. He lost the ability to walk during his fourth decade. P14 had elbow contractures and spinal stiffness mimicking Emery–Dreifuss muscular dystrophy (EDMD), but there was no cardiac involvement. P13 presented with motor delay, had distal contractures, high CK, and dystrophic biopsy, and he lost walking in adulthood. P19 and P20 were siblings and showed predominant periscapular wasting, muscle weakness, and elevated CK levels. P20 was more progressive and severe. He lost his ability to walk at puberty and developed cardiac involvement.

Globally, the cohort often showed respiratory symptoms, and 7/17 underwent respiratory function tests. All patients had normal cognitive development, and no patient died in postnatal life.

### 2.3. Morphological Data

A muscle biopsy was performed for 17 patients, and the results were heterogeneous.

Two biopsies obtained conjunctive tissue with no muscle fibers identified (failed), all of them from patients with arthrogrypotic phenotypes.

The analyses of the 15 *TTN* patients with available muscle biopsies revealed dystrophic changes in four of them, two in patients with progressive weakness and high CK levels (P1 and P13), including cardiac involvement in P1. Myopathic non-specific features were observed in six patients with non-progressive variable phenotypes. Core or minicore structures on NADH staining were reported in three cases. While cores were found in patients with neonatal congenital hypotonia and periscapular weakness (P19 and 20), one of the patients achieved a progressive course during childhood (P20). In contrast, minicores and vacuoles were identified in patients with arthrogryposis without a progressive course (P10, P11, P12).

### 2.4. MRI Imaging

**Figure 2:**
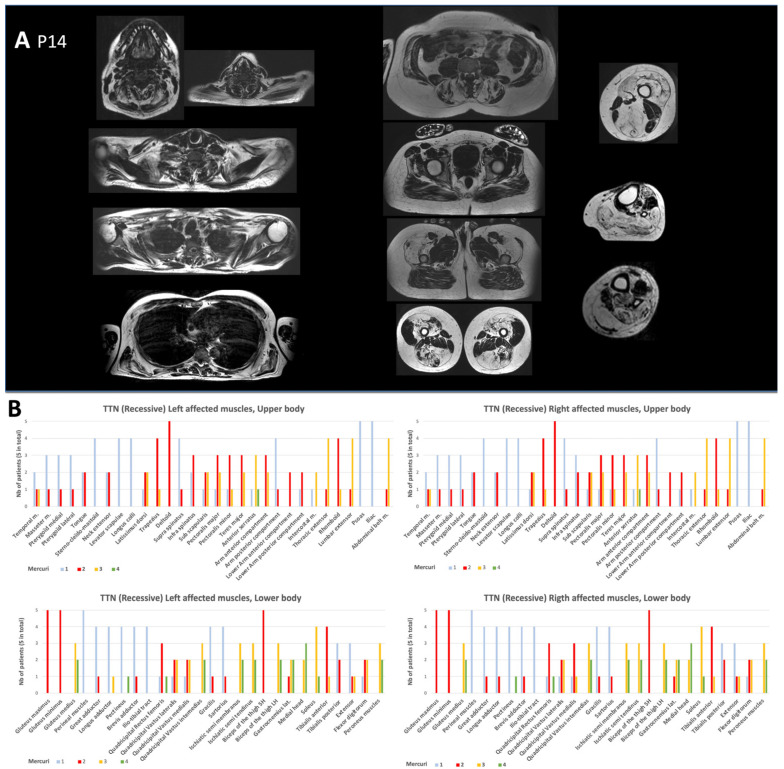
MRI data. (**A**) Patient P14 MRI analysis, selection of axial T1 weighted sections of a patient with a recessive form of titinopathy. Muscle damage with fat infiltration is disseminated; the most preserved muscles are the supraspinatus, psoas–iliacus, gluteus maximus, great and long adductors, and short head of the biceps femoris. Fat content in muscles is more pronounced in anterior and posterior thighs and legs. (**B**) Assessment of muscular damage to all four limbs using the Mercuri scale. Fatty replacement varies widely from muscle to muscle and from patient to patient. However, involvement predominates in proximal muscles: in the upper limbs, the deltoids, trapezius, rotator, and scapula fixator muscles are the most affected, whereas in the lower limbs, involvement predominates in the glutei muscles, in the thighs and legs, although the sartorius and gracilis muscles are spared.

Muscle imaging analysis showed variable and heterogeneous features, with no selectively affected muscles. The texture and signal abnormalities showed a gradation, as observed in many congenital myopathies or muscular dystrophies (scores ranging from 2 to 4 at the Mercuri scale) (Figure 2).

### 2.5. Genomic, Transcript, and Protein Studies (Table S2)

Within the twenty patients carrying two *TTN* variants, five patients had both variants in MTT-only exons (P10 homozygous variant, P17, P18, P13 and P5). The variants identified in constitutively expressed exons were in genomic regions coding for the I-band (n = 7 variants), A-band (n = 8), and M-band (n = 1) (Table S2).

**Figure 3:**
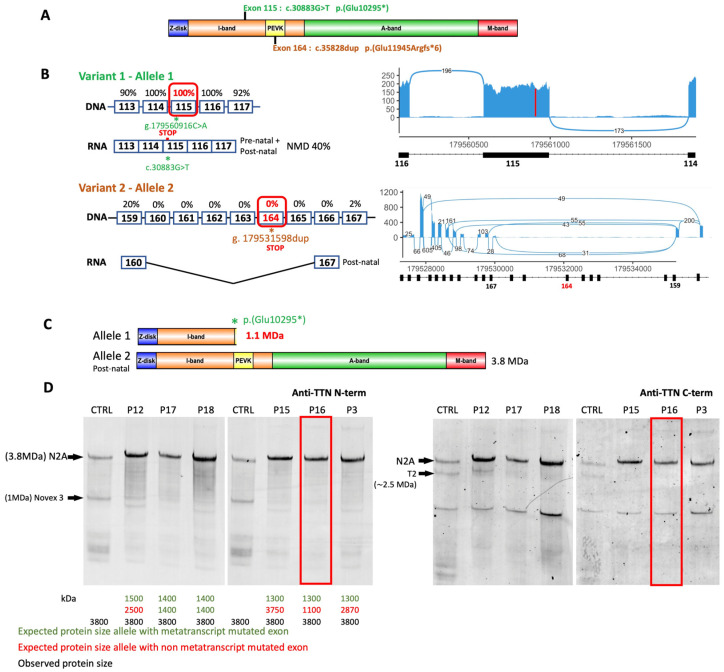
Molecular analysis of patient P16 and WBs of all available biopsies. (**A**) Localization of the variants identified in patient P16 on the *TTN* gene represented with its domains, Z-disk, I-band containing the PEVK domain, A-band, and M-band. The variants are localized in the I-band, including the variant of exon 164 in the PEVK domain. (**B**) Transcript analyses in the regions of the mutated exons. Exon inclusion rates in transcripts reported in Savarese et al., 2018 [6] are noted as percentages above DNA exons, and RNA splicing with exon inclusion is described. RNA-seq results are represented by a sashimi plot (right panel). Exon 115-carrying variant 1 is expressed in 100% of the transcripts. Exon 164 carrying variant 2 is skipped in mature N2A transcripts. (**C**) Schematic representation of the proteins theoretically translated by each allele with the predicted sizes. Allele 1 should produce a protein of about 1.1 MDa, whereas allele 2, with a variant in a metatranscript exon, should not express the variant and should translate a normal-sized protein of 3.8 MDa. (**D**) Western blot analyses of patients with *TTN* truncating variants and available muscle biopsies. Anti-TTN antibodies against the N-terminal part (Sigma1400284; left panel) and against the C-terminal part (M10.1; right panel). The analysis was performed in patients P15, P16, P3, P17, and P12. Alleles carrying frameshift variants can produce truncated proteins whose theoretical sizes have been calculated: in green, the theoretical sizes of proteins from variants in exons located in the metatranscript (if they are not skipped from N2A transcripts), and in red, the theoretical sizes of proteins from variants in exons expressed constitutively. The size of the observed titin protein is indicated in black. No titin truncated protein of 1.1 MDa is visible on the blot for P16.

In 12 patients, transcript studies were carried out on muscle biopsies by RNA-seq. Prediction data and RNA-seq results for the variants studied are presented in Supplementary Table S2. For the variants in MTT-only exons (n = 5), results on transcripts were not evaluable since the corresponding exon was skipped. P16 has two variants, confirmed to be in trans (Figure 3A). The c.30883G>T, p.(Glu10295*) truncating variant on exon 115 (I-band) was detected in the mRNA of 51 reads out of 172 total reads, suggesting partial degradation of the mutated transcript by NMD. The second variant, c.35828dup, p.(Glu11945Argfs*6), was on exon 164 and excluded from N2A transcripts (3B). A western blot (WB) on agarose gel did not detect the presence of a 1.1 MDa truncated protein corresponding to the c.30883G>T, p.(Glu10295*) variant, and there was no significant reduction in the amount of normal protein (Figure 3C,D). For variants located on splice sites, RNA-seq analyses revealed several populations of transcripts resulting from several splicing consequences: exon skipping, activation of cryptic splice sites, and most surprisingly, frequent intron retention (Table S2). P6, a fetus that died before birth in association with a multiple arthrogryposis diagnosis, carried the c.39974-11T>G variant in exon 213 that was previously described in patients with autosomal recessive arthrogryposis multiplex congenita and myopathy, which is associated with a second variant c.37543+1G>A, in metatranscript intron 184. The latter splice variant is predicted to shift the donor splice site of exon 183 (Splice AI DG: 0.82, DL: 0.97). No muscle biopsy was available for RNA-seq analysis.

RNA-seq analyses are also of great value for identifying the pathogenic consequences of missense variants on splicing. P2 had a nonsense variant c.26452C>T, p.(Gln8818*) in exon 91 and a missense variant in trans c.36285C>G p.(His12095Gln) in exon 169. This latter missense variant was predicted to result in the loss of the wild-type acceptor splice site and the activation of a cryptic splice acceptor site 5 bp downstream (SpliceAI AG: 0.89), shifting the reading frame. RNA-seq confirmed these consequences on transcripts (Table S2), enabling us to reclassify this missense variant from VUS to pathogenic according to the ACMG classification (PPS3 PM2 PM3 PP1 PP3).

The titin protein was analyzed by WBs for patients P15, P16, P3, P17, P18, and P12 carrying a truncating variant, but failed to detect any truncated proteins (Figure 3D).

## 3. Discussion

We report, in our study, 20 additional patients with antenatal or early postnatal onset of neuromuscular signs, characterized by the identification of at least one pathogenic or likely pathogenic *TTN* variant in an MTT-only exon. This cohort presented with diverse phenotypes, including arthrogryposis multiplex congenita, congenital myopathy, and muscular dystrophy, reflecting, with the previous studies of 55 cases [7,8,9,10,11,12,13,14,15,16,17,18], the heterogeneity of early-onset titinopathies. Identifying pathogenic variants in MTT-only exons may inform prognosis. This aspect requires continued documentation of patient samples in long-term studies. For patients with morphological analyses, the structural features were also very heterogeneous, from failed samples in cases of arthrogryposis, to centronuclear, core-like or fiber size disproportion in those patients with congenital myopathy phenotypes, ending with more dystrophic features. The morphological analysis did not show up defined groups of patients with specific characteristics. Our findings underscore the importance of including *TTN* in genetic testing panels for a wide range of neuromuscular disorders, particularly in patients presenting with early-onset myopathies and arthrogryposis.

A study including genetic analysis of 315 patients with an initial phenotype of arthrogryposis shows the presence of pathogenic *TTN* variants in about 8% of the resolved cases, making *TTN* the predominant gene causing arthrogryposis [12]. In this study, some patients have compound heterozygotes for truncating variants located in exons not belonging to the PEVK domain [8,11,12]. The study by Di Feo et al. shows that variants in exon 359 of *TTN* appear to be particularly associated with arthrogryposis phenotypes [17]. Therefore, even in a case of congenital multiminicore myopathy associated with arthrogryposis and mutation absence in the RYR1 [19], or even genes involved in congenital myasthenia or myopathy, analysis of the *TTN* gene and, in particular, MTT-only exons is highly recommended.

Due to the size of the gene and its molecular and transcriptional complexity, the diagnosis of a titinopathy is not as straightforward. The pathogenic effects of *TTN* variants on transcripts, as well as on protein quantity, size, and functionality [20], need to be assessed as accurately as possible. We identified 31 *TTN* variants, including 20 in an MTT-only exon located in the PEVK domain at the I-band. The location of the other variants was not specific to a particular domain. Nine variants were located within the repeated regions of *TTN*. The localization of variants in repeated areas of *TTN* is crucial for accurate molecular diagnosis of patients and for the detection of carrier relatives. To overcome the peculiarity of NGS mapping in repeated domains, we had previously implemented a relocation strategy, with the aim of precisely locating variants within the repeats using MinION (Oxford Nanopore Technologies, Oxford, UK) long-read technology. This approach was successful in confirming the location of the variants in the *TTN* repeat areas for five patients [21]. In addition, the MinION technology helps to define patient haplotypes; for example, it helped to determine that variants in exons 195 and 199 of patient P9 were on the same allele, whereas those in the same exons were on different alleles in P17.

The interpretation of variants in MTT-only exons is particularly delicate. The documentation of as many patients as possible helps in understanding the pathophysiology of congenital titinopathies due to these variants. RNAseq analyses are essential to evaluate the level of expression in variants in MTT-only exons, to assess the level of nonsense-mediated mRNA decay NMD, and to visualize the consequences of splicing variants. Our study provides further evidence that the NMD mechanism exists but is incomplete in truncated titin transcripts. We found that certain variants led to incomplete NMD, resulting in residual expression of truncated proteins, which may partially explain the degree of severity of the phenotype. However, we do not have sufficient data to interpret the importance of the contribution of NMD in the phenotype severity of these patients. Concerning consequences of variants on splicing, our results show that, in addition to affecting the canonical splice sites, most of these variants result in retention of the concerned introns in at least part of the transcript (P10, P12 and P5). Another challenge is to understand the effect of these mutations while considering the timeline expression of specific *TTN* exons during development. There is little data on affected embryos and fetuses to understand and analyze the expression of exons during this period. Previous descriptions of patients with congenital titinopathies due to variants in MTT-only exons suggest that a defect in titin in the prenatal period may be responsible for some congenital forms of the severe clinical picture at birth. The relative stability or improvement of symptoms observed in some patients after birth may be attributable to the presence of a normal protein expressed by the N2A isoform [7]. No cardiac involvement was disclosed in patients. This could be due to a lack of expression of MTT-only exons in the N2B isoform, predominant in cardiac muscle. Our patient cohort seems to show that the initial skeletal phenotype is severe but seems to improve during childhood in some cases. However, it was not possible to have homogeneous and precise data concerning patients’ evolution. This aspect, and the lack of longitudinal follow-up data, limits our ability to assess the long-term evolution of the disease in this cohort. Also, having a larger sample of patients would allow us to refine the genetic data and attempt to make a more precise genotype correlation with the clinical data of the patient subgroups. In the future, the study of titinopathies could become even more complex with the discovery of digenic variants. A study showed that the association between variants on the *TTN* and *RBM20* genes was responsible for a cardiomyopathy phenotype [22], and even more recently, the association of *TTN* and *SRPK3* variants was associated with a skeletal myopathy phenotype [23].

In conclusion, this report of new pediatric and adult cases, together with functional characterization work through RNA-seq analyses, will help to improve molecular diagnoses and provide a better understanding of the pathophysiology of these titinopathies. Future studies should focus on expanding the cohort size to better capture the full spectrum of titinopathies, as well as conducting long-term follow-ups to assess disease progression. Additionally, animal models expressing mutations in MTT-only exons would be invaluable for understanding the developmental impact of these variants and for testing potential therapeutic approaches.

## 4. Materials and Methods

### 4.1. Patients and TTN Variants

The study was approved by the ethical guidelines delivered by our institutions for clinical studies in compliance with the Helsinki Declaration. Patients or parents have given their written informed consent for the genetic analysis according to French legislation (Comité de Protection des Personnes OUEST 6-CPP1128HPS3 IDRCB-2018-AO2287-48). Patients were included in the study based on the following criteria: congenital myopathy phenotype and/or neonatal hypotonia, arthrogryposis or distal contractures, identification of at least one *TTN* variant in an MTT-only exon, and absence of other etiology of disease.

*TTN* (NM_001267550.1) variants were previously identified by next-generation sequencing (NGS), targeting exons and exon–intron junctions of gene panels or the exome, as previously reported [24,25]. The pathogenicity of variants was assessed through a set of criteria reported by Zenagui et al., 2018 [24], according to the American College of Medical Genetics and Genomics (ACMG) guidelines and reported in Table S1 [26]. In the present work, an additional tool was used, MPA software version 1.1 [27], that aggregates bioinformatic prediction tools for missense variants and provides a 0 to 10 prioritization score, validated for use in neuromuscular genes, in particular the large and complex *TTN* and NEB genes, accessible from https://mobidetails.iurc.montp.inserm.fr/MD (accessed on 1 July 2024) [28].

Prediction of the splicing variant effects were scored with SpliceAI [29]. In addition, to consider the complexity of skeletal transcripts of *TTN* and account for the fact that some exons are only expressed in the metatranscript (“MTT-only exons”), we systematically referred to the recent study reported by Savarese et al., 2018 [6].

### 4.2. Muscle Morphological Analyses and Immunohistochemistry

Open muscle biopsies were performed, and the samples were frozen and fixed following previously described standardized methods. They were then analyzed with histochemical and histoenzymatic techniques with light and electron microscopy using standard techniques [30,31].

### 4.3. Transcript Analysis

mRNAs were extracted from 20 mg of the muscle biopsy samples and prepared as described in [20]. Invitrogen Superscript II reverse transcriptase was then used to reverse-transcribe 2 μg of RNA. RNA sequencing was performed by IntegraGen SA (Evry, France) using the protocol with poly (A) selection of mRNA (Illumina (San Diego, CA, USA) TruSeq^®^ Stranded Total RNA Library Prep). Paired-end 75-bp sequencing was performed on the Illumina NovaSeq6000^®^ platform, as described in [32]. RNA-seq generated 88 million reads. RNA sequence alignment was performed using ultrafast universal RNA-seq aligner (STAR) software version 2.7.10b [33] and analyzed using Integrative Genomics Viewer (IGV) [34] targeted on titin transcripts. Transcript sashimi plot representations were made with ggsashimi [35]. The schematic images in Figure 1 and Figure 3 were created using Illustrator for Biological Sequences version 1.0.

### 4.4. Western Blot Analysis (WB)

Protein solubilization and western blot procedures were fully described in [20]. Protein solubilization from muscle biopsies was prepared as previously described [36]. For the full-length titin WB, 5 μL of protein extract was loaded on an SDS-agarose 0.8% gel prepared as previously reported [37] and run for 3 h 20 at 15 mA. The protein transfer onto the PVDF membrane was performed using wet transfer Trans-Blot cell from Bio-Rad (Bio-Rad, Hercules, CA, USA) at 400 mA for 3 h. Membranes were subsequently incubated in Odyssey blocking buffer, washed, and incubated overnight at 4 °C in Odyssey blocking buffer and PBS-specific primary antibodies: anti-titin specific for either the C-terminal part M10-1 (rabbit; 1:1000; kindly provided by Dr. Isabelle Richard [38]) or the N-terminal part (mouse; 1:1000; Sigma SAB1400284). Membranes were washed and incubated for one hour with donkey anti-mouse IR Dye 800 CW (1:30,000) or donkey anti-rabbit IR Dye 680RD (1:30,000). Membranes were then washed, dried, and scanned with the Odyssey Infrared Imaging System.

### 4.5. Muscle Imaging

Muscle imaging studies were available in 13 index cases. One patient was scanned using computerized tomography (CT). The remaining 13 had MRI examinations, 11 by whole-body MRI (“WBMRI”) techniques. All partial and insufficient qualitative MRI examinations and CT scanner images were excluded from the global WBMRI analysis.

Two types of MRI machines were used: the 1.5 and 3 Tesla. Images were analyzed with classical T1 and T2 weighting, fat saturation sequences, or by the T2 Dixon technique [39].

Mercuri scoring was performed in each study using the T1-weighted or fat image of the T2 Dixon axial slices and was analyzed to search for patterns and particular profiles.

The Mercuri classification is divided into four stages [40]: 1 = normal, 2 = fat replacement less than 30%, 3 = fat replacement ratio estimated to be between 30% and 60%, and 4 = fat replacement in muscle greater than 60%.

## 5. Conclusions

This work reinforces the involvement of titin in arthrogryposis and highlights the importance of the role of metatranscripts in these diseases. Further work is required to better understand the pathophysiological mechanisms.

## Figures and Tables

**Table 1:** Clinical phenotype and genetics.

Family ID	Patient ID	Sex	Phenotype	Variant 1	Exon_Region (Allele 1)	Variant 2	Exon_Region (Allele 2)	Confirmed Mode of Inheritance?	Segregation Results
1	1	M	LGMD-cardio	c.35713+1del	ex162_I-band	c.7120C>T p.(Gln2374*)	ex32_I-band	No	Parents not tested, brothers and sister with the same variants
2	2	Fo	AMC	c.36285C>G p.(His12095Gln)	ex170_I-band	c.26452C>T p.(Gln8818*)	ex92_I-band	Yes	Compound heterozygous
3	3	M	AMC	c.37138G>T p.(Glu12380*)	ex180_I-band	c.77667_77685del p.(Asp25889Glufs*58)	ex327_A-band	Yes	Compound heterozygous
4	4	F	AMC	c.38737G>T p.(Glu12913*)	ex199_I-band	c.87019_87022del p.(Glu29007Ilefs*16)	ex328_A-band	Yes	Compound heterozygous
5	5	F	AMC	c.36044-2A>G	in166_I-band	c.49649-2A>G	In265_A-band	Yes	Compound heterozygous
6	6	Fo F	AMC	c.37543+1G>A	In184_I-band	c.39974-11T>G	In213_I-band	Yes	Compound heterozygous
7	7	F	AMC	c.38421_38437delinsC p.(Pro12808Lysfs*134)	ex195_I-band	c.102364G>T p.(Gly34122*)	ex359_M-band	Yes	Compound heterozygous
7	8	Fo	AMC	c.38421_38437delinsC p.(Pro12808Lysfs*134)	ex195_I-band	c.102364G>T p.(Gly34122*)	ex359_M-band	Yes	Compound heterozygous
8	9	M	Axial–distal arthrogryposis	c.38437A>T p.(Lys12813*)c.38791+1G>T	ex195_I-bandin199_I-band	c.17521_17522delAC p.(Thr5841Phefs*4)	ex60_I-band	Yes	Compound heterozygous
9	10	M	Axial–distal arthrogryposis	c.36400A>T p.(Lys12134*)	ex171_I-band	c.36400 A>T p.(Lys12134*)	ex171_I-band	Yes	Homozygous variants
10	11	M	Axial–distal arthrogryposis	c.37155_37159del p.(Lys12385Asnfs*6)	ex180_I-band	c.30883G>T p.(Glu10295*)	ex115_I-band	Yes	Compound heterozygous
11	12	F	Axial–distal arthrogryposis	c.40011delA p.(Glu13338Asnfs*51)	ex214_I-band	c.56648-1G>A	in291_A-band	Yes	Compound heterozygous
12	13	M	Congenital muscular dystrophy	c.38660delA p.(Lys12887Argfs*60)	ex198_I-band	c.35678C>G p.(Thr11893Ser)	ex162_I-band	No	
13	14	M	Distal dystrophic EDMD	c.35756delC p.(Pro11919fs*51)	ex163_I-band	c.35756delC p.(Pro11919fs*51)	ex163_I-band	Yes	Homozygous variants
14	15	M	Neo Hypot Cong Myop	c.35828dup p.(Glu11945Argfs*6)	ex164_I-band	c.99511del p.(Tyr33171Ilefs*4)	ex356_A-band	Yes	Compound heterozygous
15	16	M	Neo Hypot Cong Myop	c.35828dup p.(Glu11945Argfs*6)	ex164_I-band	c.30883G>T p.(Glu10295*)	ex115_I-band	No	Mother heterozygous for allele 2, father not tested
16	17	F	Neo Hypot Cong Myop	c.38386G>T p.(Glu12796*)	ex195_I-band	c.38767A>T p.(Lys12923*)	ex199_I-band	Yes	Compound heterozygous
16	18	F	Neo Hypot Cong Myop	c.38386G>T p.(Glu12796*)	ex195_I-band	c.38767A>T p.(Lys12923*)	ex199_I-band	Yes	Compound heterozygous
17	19	M	Neo Hypot Cong Myop	c.35713+3A>G	in162_I-band	c.53918delG p.(Gly17973fs*18)	ex281_A-band	Yes	Compound heterozygous
17	20	M	Neo Hypot Cong Myop	c.35713+3A>G	in162_I-band	c.53918delG p.(Gly17973fs*18)	ex281_A-band	Yes	Compound heterozygous

LGMD: Limb-girdle muscular dystrophy, AMC: arthrogryposis multiplex congenita, Neo Hypot Cong Myop: neonatal hypotonia congenital myopathy, EDMD: Emery–Dreifuss muscular dystrophy.

## Data Availability

The original contributions presented in this study are included in the article and Supplementary Materials; further inquiries can be directed to the corresponding authors.

## Supplementary Materials

The following supporting information can be downloaded at: https://www.mdpi.com/article/10.3390/ijms252312994/s1.
