# Supplementary material for: Congenital Titinopathies Linked to Mutations in TTN Metatranscript-Only Exons
Source: Int J Mol Sci. 2024 Dec 3;25(23):12994. doi: 10.3390/ijms252312994 (PMC11641062; doi:10.3390/ijms252312994)
Supplement: Supplementary file 1 [file ijms-25-12994-s001.zip › PERRIN et al_List of investigators.pdf]

List of investigators

Valérie Biancalana[1,2], Ana Maria Navarro[3,4], Florence Esselin[5], Marie-Christine Arné-Bes[6], Vincent Fabry[7], Anne-Laure Bedat Millet[8], Anne Claire Bréhin[9], Ana Camacho Salas[10], Claude Cances[11], Jean Baptiste Davion[12], Julien Durigneux[13], Léonard Féasson[14], Ana Ferreiro[15,16], Martha Gomez Garcia de la Banda[17], Arnaud Isapof[18], Médéric Jeanne[19,20], Pascal Laforet[21], Annie Laquerriere[22], Johann Bohm[23], France Leturcq[24], Franck Letournel[25], Edoardo Malfatti[26], Pascale Marcorelles[27,28], Xénia Latypova[29,30], Arnaud Molin[31], Juliette Nectoux[24], Marie-Christine Nougues[32], Florence Petit[33], Marguerite Preud'homme[34], John Rendu[29], Isabelle Richard[35], Vincent Tiffreau[36], Sandra Whalen[37], Christian Jorgensen[38]

[1] Laboratoire de Diagnostic Génétique, Université de Strasbourg, 67084 Strasbourg, France.
[2] Laboratoire de Diagnostic Génétique, Faculté de Médecine, CHRU, Nouvel Hôpital Civil, 1 place de l'Hôpital, 67091, Strasbourg, France
[3] Laboratoire de Génétique Moléculaire, Centre Hospitalier Universitaire de Montpellier, 34093 Montpellier, France.
[4] PhyMedExp, Université de Montpellier, INSERM, CNRS, 34093 Montpellier, France.
[5] Explorations Neurologiques et Centre SLA, Centre de Référence des Maladies Neuromusculaires AOC (Atlantique-Occitanie-Caraïbe), Centre Hospitalier Universitaire de Montpellier, 34295 Montpellier, France.
[6] Explorations Neurophysiologiques, Centre SLA, Centre de référence de pathologie neuromusculaire, CHU Toulouse, France.
[7] Explorations Neurophysiologiques, Centre SLA, Centre de référence de pathologie neuromusculaire, CHU Toulouse, France.
[8] Nord/Est/Ile de France Neuromuscular Reference Center. Rouen University Hospital, Rouen, France.
[9] Department of Genetics, Normandy Centre for Genomic and Personalized Medicine, Normandie University, UNIROUEN, Inserm U1245 and Rouen University Hospital, F 76000, Rouen, France.
[10] Sección de Neurología Infantil, Servicio de Neurología, Hospital Universitario 12 de Octubre, Universidad Complutense de Madrid, España.
[11] Service de Neuropédiatrie, Centre Hospitalier Universitaire de Toulouse, Centre de référence des Maladies Neuromusculaires AOC (Atlantique-Occitanie-Caraïbe), 31059 Toulouse, France.
[12] Centre de Référence des Maladies Neuromusculaires Nord/Est/Ile de France, Service de Neuropédiatrie, Hôpital Salengro CHU Lille, Lille, France.
[13] Centre de Référence des Maladies Neuromusculaires AOC, CHU d'Angers, Angers, France.
[14] Université Jean Monnet Saint-Etienne, Laboratoire Interuniversitaire de Biologie de la Motricité, EA 7424, F-42023 Saint-Etienne, France; Unité de Myologie, Service de Physiologie Clinique et de l'Exercice, Centre Référent Maladies Neuromusculaires Euro-NmD, CHU de Saint-Etienne, France.
[15] APHP, Centre de Référence des Pathologies Neuromusculaires Nord-Est-Ile de France, Service de Neuromyologie, Institut de Myologie, GHU Pitié-Salpêtrière, Paris, France.
[16] Basic and Translational Myology laboratory, Université de Paris BFA, UMR 8251, CNRS, Paris, France.
[17] APHP, GH Université Paris-Saclay, Neuromuscular Center, Child Neurology and ICU Department, Raymond Poincare Hospital, Garches, France.
[18] Centre de Référence des Maladies Neuromusculaires Nord/Ile de France/Est, Service de Neuropédiatrie, Hôpital Trousseau, APHP, Paris, France.
[19] UMR 1253, iBrain, Université de Tours, Inserm, 37032 Tours, France.
[20] Department of Genetics, University Hospital of Tours, Tours, France
[21] Nord/Est/Ile de France Neuromuscular Reference Center, PHENIX FHU, Hôpital Raymond-Poincaré, AP-HP. INSERM U1179, Garches, France.
[22] Department of Pathology, Normandy Centre for Genomic and Personalized Medicine, Normandie University, UNIROUEN, Inserm U1245 and Rouen University Hospital, F 76000, Rouen, France.
[23] Institut de Génétique Et de Biologie Moléculaire Et Cellulaire (IGBMC), Inserm U 1258, CNRS UMR 7104, Université de Strasbourg, Illkirch, France

[24] Service de Médecine Génomique des Maladies de Système et d'Organe, Hôpital Cochin, AP.HP.CUP, Paris, France.
[25] Laboratoire de neurobiologie et neuropathologie, Centre Hospitalier Universitaire d'Angers, Angers, France.
[26] UPEC, Paris Est University, IMRB INSERM U955, APHP, Centre de référence neuromusculaire, HU Henri Mondor, Créteil, France.
[27] Department of Pathology, Brest University Hospital, Brest, France.
[28] Laboratory of Neurosciences of Brest, Faculté de Médecine et des Sciences de la Santé, Université de Bretagne Occidentale, Brest, France.
[29] Université Grenoble Alpes, Inserm, U1216, CHU Grenoble Alpes, Grenoble Institute of Neurosciences, 38000 Grenoble, France.
[30] Department of Genetics, Robert-Debré University Hospital, Assistance Publique-Hôpitaux de Paris, France - Université de Paris, UMR7216, Epigenetics and Cell Fate, 75013, Paris, France.
[31] Normandy University, UNICAEN, Caen University Hospital, Department of Genetics, Reference Center of Rare Diseases of Calcium and Phosphorus Metabolism, EA 7450 BioTARGen, Caen, France.
[32] Pediatric Neurology Department, Reference Centre for Neuromuscular Diseases, Armand Trousseau Hospital, APHP, Sorbonne University, 26, avenue du Docteur Arnold Netter, 75012 Paris, France.
[33] CHU Lille, Clinique de Génétique Guy Fontaine, Lille, France.
[34] Centre de référence Nord Est Ile-de-France, Service de rééducation fonctionnelle, CHU de Lille
[35] INTEGRARE, Genethon, Inserm, Université Evry, Université Paris-Saclay, 91002 Evry, France.
[36] Physical and Rehabilitation Medicine Unit, University Hospital, Lille, France, URePSSS (Pluridisciplinary Research Unit: Sports, Health, Society) EA, 7369, Lille University.
[37] UF de génétique Clinique et Centre de Référence Anomalies du Développement et Syndromes Malformatifs, Assistance Publique-Hôpitaux de Paris (APHP) Sorbonne Université, Hôpital Armand Trousseau, ERN-ITHACA, Paris, France.
[38] IRMB, Univ Montpellier, INSERM, Montpellier, France.
